# Effects of interdot hopping and Coulomb blockade on the thermoelectric properties of serially coupled quantum dots

**DOI:** 10.1186/1556-276X-7-257

**Published:** 2012-05-16

**Authors:** David M T Kuo, Yia-Chung Chang

**Affiliations:** 1 Department of Electrical Engineering and Department of Physics, National Central University, Chungli, 320, Taiwan; 2 Research Center for Applied Sciences, Academic Sinica, Taipei, 115, Taiwan

## Abstract

We have theoretically studied the thermoelectric properties of serially coupled quantum dots (SCQDs) embedded in an insulator connected to metallic electrodes. In the framework of Keldysh Green’s function technique, the Landauer formula of transmission factor is obtained using the equation of motion method. Based on such analytical expressions of charge and heat currents, we calculate the electrical conductance, Seebeck coefficient, electron thermal conductance, and figure of merit (ZT) of SCQDs in the linear response regime. The effects of interdot hopping and electron Coulomb interactions on ZT are analyzed. We demonstrate that ZT is not a monotonic increasing function of interdot electron hopping strength (*t*_*c*_). We also show that in the absence of phonon thermal conductance, SCQD can reach the Carnot efficiency as *t*_*c*_approaches zero.

## Review

### Introduction

**Figure 1:**
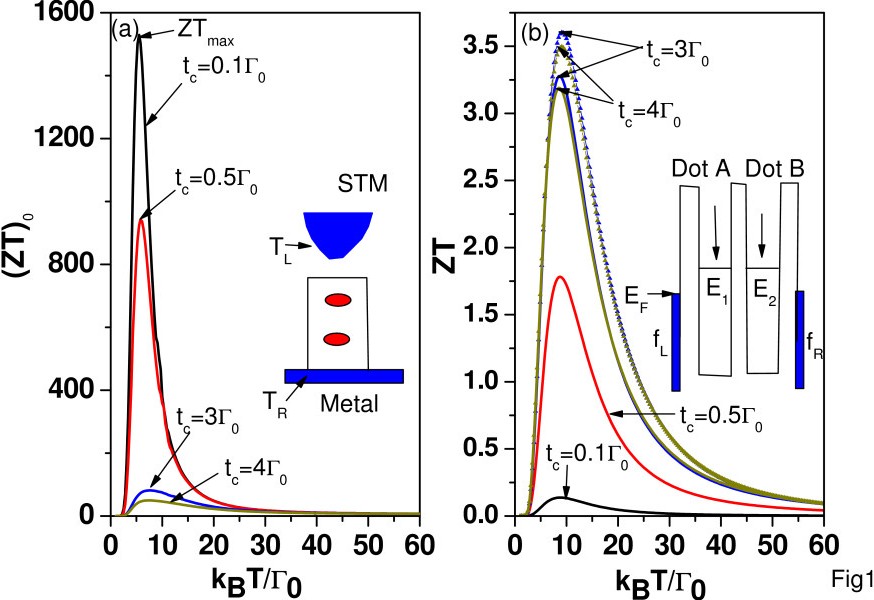
**Functions of temperature.** (**a**) (ZT)_0_ and (**b**) ZT as functions of temperature for various interdot hopping strengths (*t*_*c *_= 0.1,0.5,1,3, and 4*Γ*_0_). *E*_*ℓ *_=* E*_*F*_ + 30*Γ*_0_, *U*_*ℓ *_= 30* Γ*_0_, *U*_*ℓ*,*j *_=10* Γ*_0_, and *Γ*_*L *_=* Γ *=* Γ*_0_. STM, scanning tunneling microscope.

Recently, many considerable studies have been devoted to seeking efficient thermoelectric materials with the figure of merit (ZT) larger than 3 because there exist potential applications of solid-state thermal devices such as coolers and power generators [1-6]. Some theoretical efforts have pointed out that a single quantum dot (QD) junction system can have a very impressive ZT in the absence of phonon conductance [7-9]. However, in practice, it is difficult to maintain a large temperature gradient which is needed to produce sufficient temperature difference across the nanoscale junction. To reduce the temperature gradient across the QD junction, it is essential to consider many serially coupled quantum dots (SCQDs) [1,5]. The transport property of a junction involving *N* serially coupled QDs with strong electron Coulomb interactions is one of the most challenging topics of condensed matter physics. To gain some insight, we investigate in the present paper the thermoelectric effect of serially coupled quantum dots (SCQDs) as shown in the inset of Figure 1a.

It has been shown that the transport properties of the SCQD system exhibit several interesting features, including current rectification (due to the Pauli spin blockade), negative differential conductance, nonthermal broadening of tunneling current, and coherent tunneling in the Coulomb blockade regime [10]. Although many theoretical investigations of the above phenomena have been reported, most of them did not investigate the thermoelectric properties of SCQDs [11-13]. This study investigates the ZT of a SCQD embedded in a semiconductor nanowire with small phonon thermal conductance [4]. It is expected that the SCQD system has a potential to enhance the ZT of nanowires. Here, we consider nanoscale semiconductor QDs, in which the energy level separations are much larger than their on-site Coulomb interactions and thermal energies. Thus, only one energy level for each quantum dot needs to be considered. A two-level Anderson model [13] is employed to simulate the SCQD junction system.

### Theoretical model

Using Keldysh-Green’s function technique [13], the charge and heat currents of SCQD connected to metallic electrodes are given by

(1) $$J=\frac{2e}{h}\int d\varepsilon T(\varepsilon )\left[f_{L}(\varepsilon )-f_{R}(\varepsilon )\right],\;$$

(2) $$Q=\frac{2}{h}\int d\varepsilon T(\varepsilon )(\varepsilon -E_{F}-e\Delta V)\left[f_{L}(\varepsilon )-f_{R}(\varepsilon )\right],\;$$

where $T(\varepsilon )\equiv (T_{12}(\varepsilon )+T_{21}(\varepsilon ))/2$ is the transmission factor. $f_{L=1(R=2)}(\varepsilon )=1/\left[e^{(\varepsilon -\mu_{L(R)})/k_{B}T_{L(R)}}+1\right]$ denotes the Fermi distribution function for the left (right) electrode. The left (right) chemical potential is given by *μ*_*L*_(*μ*_*R*_). *T*_*L*(*R*)_ denotes the equilibrium temperature of the left (right) electrode. *e* and *h* denote the electron charge and Planck’s constant, respectively. $T_{\ell ,j}(\varepsilon )$ denotes the transmission function, which can be calculated by evaluating the on-site retarded Green’s function (GF) and lesser GF [13]. The indices *ℓ* and j denote the *ℓ*th QD and the *j*th QD, respectively. Based on the equation of motion method, we can obtain analytical expressions of all GFs in the Coulomb blockade regime. Details are provided in [13]. The transmission function in the weak interdot limit (*t*_*c*_/*U*_*ℓ *_≪ 1, where* t*_*c*_ and* U*_*ℓ*_ denote the electron interdot hopping strength and on-site Coulomb interaction, respectively) can be recast into the following form:

(3) Tℓ,j(ε)=−2∑m=18Γℓ(ε)Γjm(ε)Γℓ(ε)+Γjm(ε)ImGℓ,m,σr(ε),

where Im means taking the imaginary part of the function that follows and

(4) $$G_{\ell ,m,\sigma }^{r}(\varepsilon )=p_{m}/(\mu_{\ell }-\Sigma_{m}).$$

*Γ*_*ℓ*=*L*(1),*R*(2)_(*ε*) denotes the tunnel rate from the left electrode to dot A (*E*_1_) and the right electrode to dot B (*E*_2_), which is assumed to be energy- and bias-independent for simplicity. *μ*_*ℓ *_=* ε*−*E*_*ℓ*_ + *iΓ*_*ℓ*_/2. We can assign the following physical meaning to Equation 3. The sum in Equation 3 is over eight possible configurations labeled by *m*. We consider an electron (of spin *σ*) entering level *ℓ*, which can be either occupied (with probability $N_{\ell ,\bar{\sigma }}$) or empty (with probability $1-N_{\ell ,\bar{\sigma }}$). For each case, the electron can hop to level *j*, which can be empty (with probability $a_{j}=1-N_{j,\sigma }-N_{j,\bar{\sigma }}+c_{j}$), singly occupied in a spin $\bar{\sigma }$ state (with probability $b_{j,\bar{\sigma }}=N_{j,\bar{\sigma }}-c_{j}$) or spin *σ* state (with probability*b*_*j*,*σ *_=* N*_*j*,*σ *_−* c*_*j*_), or a double-occupied state (with probability*c*_*j*_). Thus, the probability factors associated with the eight configurations appearing in Equation 4 become $p_{1}=(1-N_{\ell ,\bar{\sigma }})a_{j}$, $p_{2}=(1-N_{\ell ,\bar{\sigma }})b_{j,\bar{\sigma }}$, $p_{3}=(1-N_{\ell ,\bar{\sigma }})b_{j,\sigma }$, $p_{4}=(1-N_{\ell ,\bar{\sigma }})c_{j}$, $p_{5}=N_{\ell ,\bar{\sigma }}a_{j}$, $p_{6}=N_{\ell ,\bar{\sigma }}b_{j,\bar{\sigma }}$, $p_{7}=N_{\ell ,\bar{\sigma }}b_{j,\sigma }$, and $p_{8}=N_{\ell ,\bar{\sigma }}c_{j}$. *Σ*_*m*_ in the denominator of Equation 4 denotes the self-energy correction due to Coulomb interactions and coupling with level *j* (which couples with the other electrode) in configuration *m*. We have $\Sigma_{1}=t_{c}^{2}/\mu_{j}$, $\Sigma_{2}=U_{\ell ,j}+t_{c}^{2}/(\mu_{j}-U_{j})$, $\Sigma_{3}=U_{\ell ,j}+t_{c}^{2}/(\mu_{j}-U_{j,\ell })$, $\Sigma_{4}=2U_{\ell ,j}+t_{c}^{2}/(\mu_{j}-U_{j}-U_{j,\ell })$, $\Sigma_{5}=U_{\ell }+t_{c}^{2}/(\mu_{j}-U_{j,\ell })$, $\Sigma_{6}=U_{\ell }+U_{\ell ,j}+t_{c}^{2}/(\mu_{j}-U_{j}-U_{j,\ell })$, $\Sigma_{7}=U_{\ell }+U_{\ell ,j}+t_{c}^{2}/(\mu_{j}-2U_{j,\ell })$, and $\Sigma_{8}=U_{\ell }+2U_{\ell ,j}+t_{c}^{2}/(\mu_{j}-U_{j}-2U_{j,\ell })$. *E*_*ℓ*_, *U*_*ℓ*_, and* U*_*ℓ*,*j*_ denote, respectively, the energy levels of dots, intradot Coulomb interactions, and interdot Coulomb interactions. Here, $\Gamma_{j}^{m}=-2$Im* Σ*_*j *_denotes the effective tunneling rate from level *l* to the other electrode through level *j* in configuration *m*. For example, $\Gamma_{j}^{1}=-2$Im $t_{c}^{2}/\mu_{j}=t_{c}^{2}\Gamma_{j}/\left[{\left(\varepsilon -E_{j}\right)}^{2}+{\left(\Gamma_{j}/2\right)}^{2}\right]$. It is noted that $\Gamma_{j}^{m}$ has a numerator*Γ*_*j*_ for all configurations. Furthermore, $G_{\ell ,\sigma }^{r}(\varepsilon )=\overset{8}{\underset{m=1}{\sum }}G_{\ell ,m,\sigma }^{r}(\varepsilon )$ is just the on-site single-particle retarded GF for level *ℓ* as given in Equation (A16) of [13], and $G_{\ell ,m,\sigma }^{r}(\varepsilon )$ corresponds to its partial GF in configuration *m*. The transmission function written this way has the same form as Landauer’s formula for a single QD with multiple energy levels including intralevel and interlevel electron Coulomb interactions [14,15].

The probability factors of Equation 3 are determined by the thermally averaged one-particle occupation number and two-particle correlation functions, which can be obtained by solving the on-site lesser Green’s functions [13]:

(5) $$N_{\ell ,\sigma }=-\int \frac{d\varepsilon }{\Pi }\overset{8}{\underset{m=1}{\sum }}\frac{\Gamma_{\ell }f_{\ell }(\varepsilon )+\overset{m}{\underset{j}{\Gamma }}f_{j}(\varepsilon )}{\Gamma_{\ell }+\overset{m}{\underset{j}{\Gamma }}}\text{Im}\overset{r}{\underset{\ell ,m,\sigma }{G}}(\varepsilon ),$$

and

(6) $$c_{\ell }=-\int \frac{d\varepsilon }{\Pi }\overset{8}{\underset{m=5}{\sum }}\frac{\Gamma_{\ell }f_{\ell }(\varepsilon )+\overset{m}{\underset{j}{\Gamma }}f_{j}(\varepsilon )}{\Gamma_{\ell }+\overset{m}{\underset{j}{\Gamma }}}\text{Im}\overset{r}{\underset{\ell ,m,\sigma }{G}}(\varepsilon ).$$

Note that *ℓ *≠* j *in Equations 3, 5, and 6. In the linear response regime, Equations 1 and 2 can be rewritten as follows:

(7) $$J={ℒ}_{11}\frac{\Delta V}{T}+{ℒ}_{12}\frac{\mathrm{\Delta T}}{T^{2}}$$

(8) $$Q={ℒ}_{21}\frac{\Delta V}{T}+{ℒ}_{22}\frac{\mathrm{\Delta T}}{T^{2}},$$

where *ΔV *=* μ*_*L *_−* μ*_*R *_and *ΔT *=* T*_*L *_−* T*_*R*_ are the voltage and temperature differences across the junction, respectively. Thermoelectric response functions in Equations 7 and 8 are given by

(9) $${ℒ}_{11}=\frac{2e^{2}T}{h}\int d\varepsilon T(\varepsilon ){\left(\frac{\partial f(\varepsilon )}{\partial E_{F}}\right)}_{T},$$

(10) $${ℒ}_{12}=\frac{2eT^{2}}{h}\int d\varepsilon T(\varepsilon ){\left(\frac{\partial f(\varepsilon )}{\partial T}\right)}_{E_{F}},$$

(11) $${ℒ}_{21}=\frac{2\text{eT}}{h}\int d\varepsilon T(\varepsilon )(\varepsilon -E_{F}){\left(\frac{\partial f(\varepsilon )}{\partial E_{F}}\right)}_{T},$$

and

(12) $${ℒ}_{22}=\frac{2T^{2}}{h}\int d\varepsilon T(\varepsilon )(\varepsilon -E_{F}){\left(\frac{\partial f(\varepsilon )}{\partial T}\right)}_{E_{F}}.$$

Here, T(ε) and $f(\varepsilon )=1/\left[e^{(\varepsilon -E_{F})/k_{B}T}+1\right]$ are evaluated in the equilibrium condition. It can be shown that the Onsager relation ${ℒ}_{12}={ℒ}_{21}$ is preserved. These thermoelectric response functions can also be found in [7], where authors investigated the thermoelectric properties of a single QD.

If the system is in an open circuit, the electrochemical potential will form in response to a temperature gradient; this electrochemical potential is known as the Seebeck voltage (Seebeck effect). The Seebeck coefficient (amount of voltage generated per unit temperature gradient) is defined as $S=\Delta V/\mathrm{\Delta T}=-{ℒ}_{12}/(T{ℒ}_{11})$. To judge whether the system is able to generate power or refrigerate efficiently, we need to consider the figure of merit, which is given by

(13) $$\text{ZT}=\frac{S^{2}G_{e}T}{\kappa_{e}+\kappa_{\text{ph}}}\equiv \frac{{\left(\text{ZT}\right)}_{0}}{1+\kappa_{\text{ph}}/\kappa_{e}}.$$

Here, $G_{e}={ℒ}_{11}/T$ is the electrical conductance, and $\kappa_{e}=(({ℒ}_{22}/T^{2})-{ℒ}_{11}S^{2})$ is the electron thermal conductance. (ZT)_0_ represents the ZT value in the absence of phonon thermal conductance, *κ*_*ph*_. For simplicity, we assume *κ*_*ph *_=* κ*_*ph*,0_*F*_*s*_[16-18]. $\kappa_{\text{ph},0}=\frac{\Pi^{2}k_{B}^{2}T}{3h}$ is the universal phonon thermal conductance arising from acoustic phonon confinement in a nanowire [16-18], which was confirmed in the phonon wave guide [19]. The expression of *κ*_*ph *_=* κ*_*ph*,0_*F*_*s*_ with *F*_*s *_= 0.1 can explain well the phonon thermal conductance of silicon nanowire with surface states calculated by the first-principles method [16]. The dimensionless scattering factor *F*_*s *_arises from phonon scattering with surface impurities or surface defects of quantum dots [1,16]. Here, we adopt *F*_*s *_= 0.02, which is smaller than *F*_*s *_= 0.1 because QDs can enhance the phonon scattering rates and reduce phonon thermal conduction as pointed out in [1].

### Results and discussion

Here, we consider the case of identical QDs in the optimization of ZT, although it is understood that the size fluctuation of QDs can suppress ZT [13]. In Figure 1a,b we plot (ZT)_0_ and ZT as a function of temperature for various electron hopping strengths. We adopt the following physical parameters: *E*_*ℓ *_=*E*_*F*_ + 30*Γ*_0_, *U*_*ℓ *_= 30*Γ*_0_, *U*_*ℓ*,*j *_= 10*Γ*_0_, and *Γ*_*L *_=* Γ*_*R *_=* Γ *= 1*Γ*_0_. All energy scales are in the units of the characteristic energy, *Γ*_0_. In Figure 1a, we see that (ZT)_0_ increases with decreasing *t*_*c *_and diverges as *t*_*c*_ → 0. This behavior can be proved rigorously as we shall illustrate below. It implies that SCQD can reach the Carnot efficiency in the limit of extremely weak interdot coupling, if one can fully suppress *κ*_*ph*_, for example, by inserting a nanoscale vacuum layer to block the phonon heat current. Although it would be a challenging task to implement a vacuum layer between one of the electrodes and SCQD, it may be possible to test this idea out via a scanning tunneling microscopic experiment using a setup as shown in the inset of Figure 1a. In Figure 1b, we see that ZT is enhanced with increasing *t*_*c*_ until *t*_*c*_ reaches 3*Γ*_0_, and it becomes reduced for higher *t*_*c*_.

**Figure 2:**
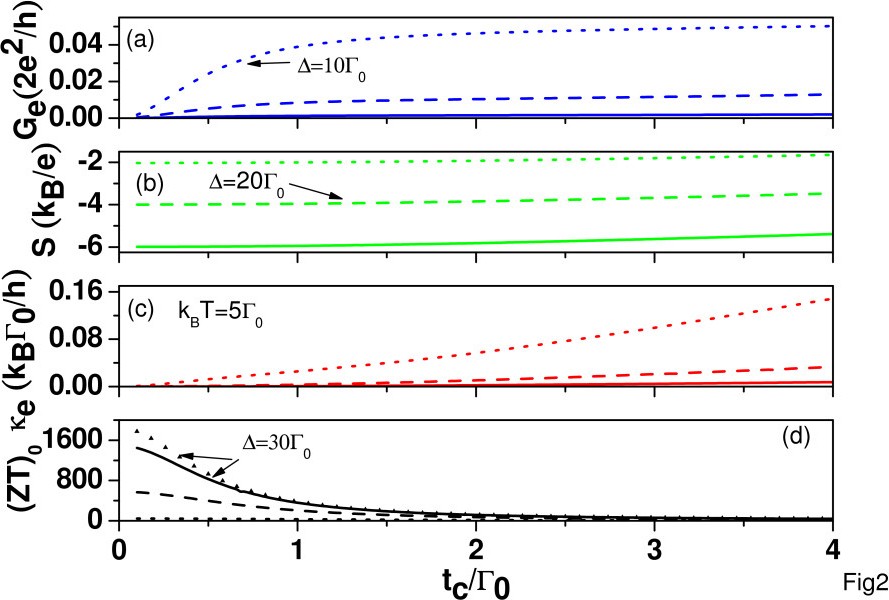
**Functions of*t***_***c***_at ***k***_***B***_***T ***= 5*Γ*_***0***_**.** (**a**) Electrical conductance (*G*_*e*_), (**b**) Seebeck coefficient (S), (**c**) electrical thermal conductance (*κ*_*e*_), and (**d**) (ZT)_0_ as functions of *t*_*c*_at *k*_*B*_*T *= 5*Γ*_0_for *Δ *= 10*Γ*_0_(dotted curves), 20*Γ*_0_(dashed curves), and 30*Γ*_0_(solid curves). Other parameters are the same as those of Figure 1.

The diverging behavior of (ZT)_0_ with respect to t_c_ is further illustrated in Figure 2d. The maximum ZT is suppressed in the presence of *κ*_*ph*_, which is much larger than *κ*_*e *_for small *t*_*c*_. The behaviors of ZT shown in Figure 1b are mostly determined by the power factor (*S*^2^*G*_*e*_). Once *t*_*c*_ is larger than 3*Γ*_0_, the reduction of* S*^2^ is faster than the increase of *G*_*e*_. This explains why the maximum ZT at *t*_*c *_= 4*Γ*_0_ is smaller than that at *t*_*c *_= 3*Γ*_0_. The location of ZT_max_ is nearly independent of *t*_*c*_, and it occurs near *k*_*B *_*T *= 8.8*Γ*_0_. For comparison, we also show the results (curves with triangle marks) for the case without electron Coulomb interactions in Figure 1b. It is seen that the maximum ZT is enhanced when we turn off the electron Coulomb interactions. Such a behavior is similar to that of a single QD with multiple energy levels [7,8]. The effect of electron Coulomb interactions is significant only for temperature between 6*Γ*_0_ and 50*Γ*_0_. Namely, the electron Coulomb interactions are negligible when *U*/(*k*_*B*_*T*)≫1or *U*/(*k*_*B*_*T*)≪1.

To further understand the behavior of ZT with respect to *t*_*c*_, we plot the electrical conductance (*G*_*e*_), Seebeck coefficient (*S*), electrical conductance *κ*_*e*_, and (ZT)_0_ as functions of *t*_*c*_ in Figure 2 for various detuning energies, *Δ*≡*E*_*ℓ*_−*E*_*F*_. When *E*_*ℓ*_ is close to the Fermi energy, *G*_*e*_ and *κ*_*e*_ are enhanced, whereas *S* and (*ZT*)_0_ are suppressed. The behavior of (ZT)_0_ at *Δ *= 30*Γ*_0_ in the absence of Coulomb interactions is also shown by the curve with triangles, which has a similar trend as the solid line. Thus, it is instructive to analyze (ZT)_0_ in the absence of Coulomb interactions. Keeping the leading order of $t_{c}^{2}$, we have ${ℒ}_{11}=\frac{2e^{2}}{hk_{B}}\frac{t_{c}^{2}}{\Gamma_{0}/2}\frac{1}{\text{cos}h^{2}(\Delta /2k_{B}T)}$, ${ℒ}_{12}={ℒ}_{21}=\frac{2e}{hk_{B}}\frac{t_{c}^{2}}{\Gamma_{0}/2}\frac{\Delta }{\text{cos}h^{2}(\Delta /2k_{B}T)}$, and ${ℒ}_{22}=\frac{2}{hk_{B}}\frac{t_{c}^{2}}{\Gamma_{0}/2}\frac{\Delta^{2}}{\text{cos}h^{2}(\Delta /2k_{B}T)}$. Therefore, $G_{e}\propto t_{c}^{2}$, *S *= −* Δ*/*eT* is independent on *t*_*c*_, and $\kappa_{e}=({ℒ}_{22}-{ℒ}_{12}^{2}/{ℒ}_{11})/T^{2}$ vanishes up to $t_{c}^{2}$. Thus, the leading order of *κ*_*e *_is $t_{c}^{4}$. This indicates that (ZT) ${}_{0}\propto 1/t_{c}^{2}$ in the limit of weak interdot hopping.

**Figure 3:**
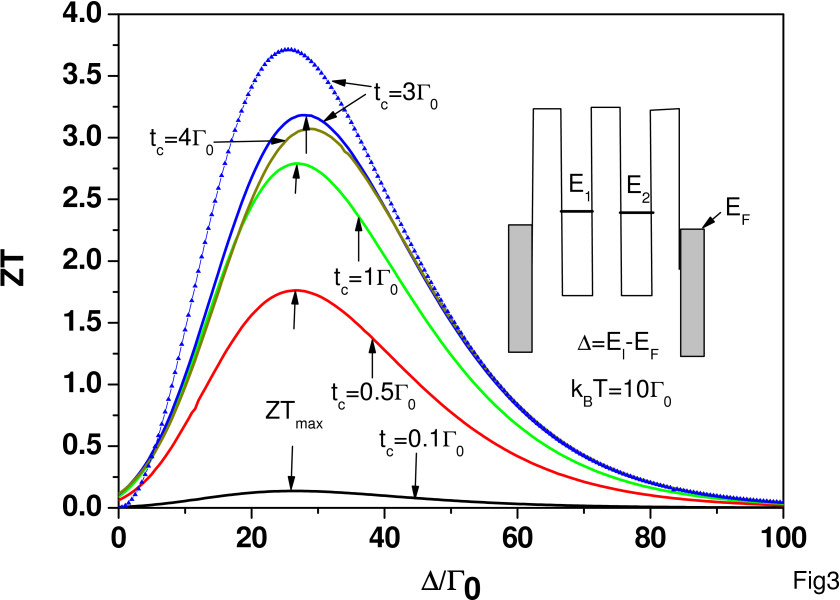
**ZT as a function of*Δ***for different electron hopping strength at ***k***_***B***_***T ***= 10*Γ*_***0***_**.** Other parameters are the same as those of Figure 1.

Figure 3 shows ZT as a function of *Δ *=* E*_*ℓ*_−*E*_*F *_for various electron hoping strengths at *k*_*B*_*T *= 10*Γ*_0_. Other physical parameters are kept the same as those for Figure 1. When *t*_*c *_= 0.1*Γ*_0_, the maximum ZT (ZT_max_) occurs at near *Δ *= 27*Γ*_0_. The peak position only shifts slightly to higher *Δ*with increasing *t*_*c*_. We have ZT_max_ = 2.79 and 3.18 for *t*_*c *_= 1*Γ*_0_ and 3*Γ*_0_, respectively. However, at *t*_*c *_= 4*Γ*_0_, we have ZT_max_ = 3.07, which is smaller than ZT_max_ for *t*_*c *_= 3*Γ*_0_. Thus, it also illustrates that ZT is not a monotonically increasing function of *t*_*c*_. We further calculated ZT as a function of *t*_*c *_for *Δ *= 10,20,30*Γ*_0_ and *k*_*B*_*T *= 10*Γ*_0_ in the presence of *κ*_*ph *_and found that again, ZT is not a monotonically increasing function of *t*_*c*_ (not shown here). We conclude that as long as *κ*_*ph *_dominates over *κ*_*e*_, the *t*_*c*_ dependence of ZT is mainly determined by the power factor *S*^2^*G*_*e*_, where the behaviors of *G*_*e*_ and *S* are similar to the results shown in Figure 2a,b. When *t*_*c*_/*Γ*_0_≤1, *G*_*e*_ increases much faster than the reduction of*S*^2^ for increasing *t*_*c*_, and the power factor slowly reaches the maximum when *t*_*c *_approaches 3*Γ*_0_. When *t*_*c *_> 3*Γ*_0_, the power factor decreases due to the fast reduction of*S*^2^ which prevails over the increase of *G*_*e*_. The curve with triangle marks is for *t*_*c *_= 3*Γ*_0_ in the absence of Coulomb interaction. We see that ZT_max_ is larger when *U*_*ℓ *_=*U*_*ℓ*,*j *_= 0. Based on the results of Figure 3, we conclude that it is important to control the detuning energy *Δ* for the optimization of *ZT*.

**Figure 4:**
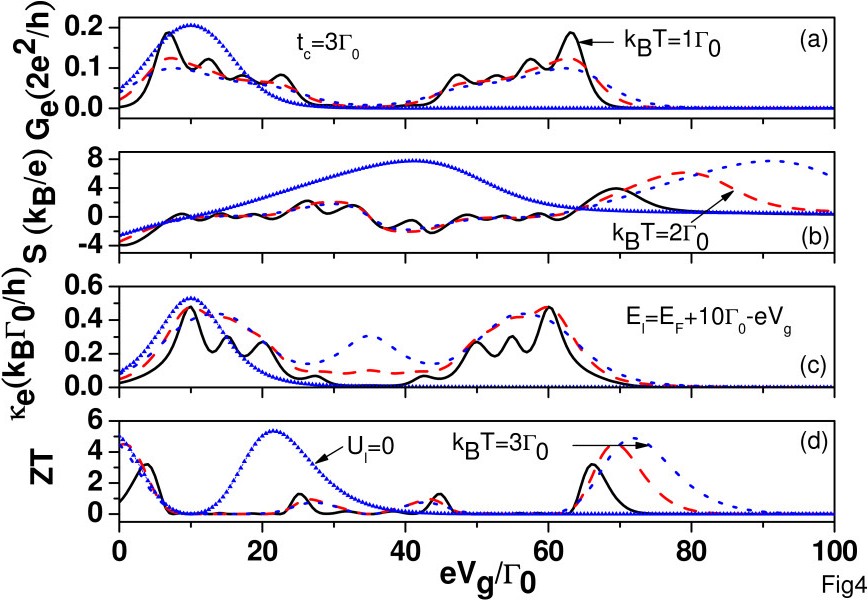
**Function of applied gate voltage.** (**a**) *G*_*e*_, (**b**) S, (**c**) *κ*_*e*_, and (**d**) ZT as a function of applied gate voltage for *k*_*B*_*T *= 1*Γ*_0_(solid), 2*Γ*_0_(dashed), and 3*Γ*_0_(dotted). *E*_*ℓ *_=* E*_*F*_ + 10*Γ*_0_ and *t*_*c *_= 3*Γ*_0_. Other parameters are the same as those of Figure 1. The curves with triangle marks are for the case without electron Coulomb interactions for *k*_*B*_*T *= 3*Γ*_0_.

In Figures 1, 2, and 3 we have considered the case with *E*_*F *_below QD energy levels. It would be interesting to investigate the case with *E*_*F*_ above the energy levels of QDs. Figure 4 shows *G*_*e*_, S, *κ*_*e*_, and ZT of an SCQD with *t*_*c *_= 3*Γ*_0_ as functions of applied gate voltage for various temperatures. Once *t*_*c*_>(*Γ*_*L*_ + *Γ*_*R*_) = 2*Γ*_0_, the eight peaks for *G*_*e*_ can be resolved at *k*_*B*_*T *= 1*Γ*_0_. These eight peaks correspond to the following resonant channels: *E*_*ℓ *_−*t*_*c*_, *E*_*ℓ*_ + *t*_*c*_, *E*_*ℓ*_ + *U*_*ℓ*,*j*_−*t*_*c*_, *E*_*ℓ*_ + *U*_*ℓ*,*j*_ + *t*_*c*_, *E*_*ℓ*_ + *U*_*ℓ*,*j*_ + *U*_*ℓ *_−* t*_*c*_, *E*_*ℓ*_ + *U*_*ℓ*,*j*_ + *U*_*ℓ*_ + *t*_*c*_, *E*_*ℓ*_ + 2*U*_*ℓ*,*j*_ + *U*_*ℓ *_−* t*_*c*_, and *E*_*ℓ*_ + 2*U*_*ℓ*,*j*_ + *U*_*ℓ*_ + *t*_*c*_, which are tuned by the gate voltage to be aligned with *E*_*F*_. These eight channels result from the four configurations of *p*_1_, *p*_3_, *p*_6_, and *p*_8_ in Equation 4. Such a result implies that SCQD with identical QDs acts as a QD with effective two levels of *E*_*ℓ *_−*t*_*c*_ and *E*_*ℓ*_ + *t*_*c *_ and satisfying Hund’s rule. These eight peaks are smeared out with increasing temperature. The sign changes of *S* with respect to the gate voltage result from the bipolar effect, i.e., the competition between electrons and holes, where holes are defined as the unoccupied states below *E*_*F*_[13]. The electronic thermal conductance (*κ*_*e*_) also exhibits eight peaks, and we noticed that the local maxima of the *κ*_*e*_ curve nearly coincide with the local minima of the *G*_*e*_ curve. We see that ZT values are still larger than 3 even when *E*_*ℓ*_is deeply below *E*_*F*_(say, at *eV*_*g *_= 70*Γ*_0_). This is attributed to the electron Coulomb interaction. To illustrate that, we also show the results with *U*_*ℓ *_=*U*_*ℓ*,*j *_= 0 at *k*_*B*_*T *= 3*Γ*_0_(see the curve with triangle marks). The oscillation of ZT in the case of *U*_*ℓ *_=*U*_*ℓ*,*j *_= 0 is attributed to the sign change of *S* at *V*_*g *_= 10*Γ*_0_. Note that *S* goes to zero at *V*_*g *_= 10*Γ*_0_, which results from the electron-hole symmetry (with *E*_*ℓ*_ + *t*_*c *_and *E*_*ℓ *_−*t*_*c*_ straddling *E*_*F*_ symmetrically). We see that ZT vanishes for *eV*_*g *_≥ 40*Γ*_0_in the absence of electron Coulomb interactions. Unlike the case of *E*_*F *_<*E*_*ℓ*_, where the finite *U* causes reduction of ZT, here, the electron Coulomb interaction leads to enhancement of ZT when *E*_*F *_>*E*_*ℓ*_.

## Conclusions

In summary, the thermoelectric properties including *G*_*e*_, *S*, *κ*_*e*_, and ZT of the SCQD junction system are investigated theoretically. We demonstrate that the Carnot efficiency can be reached when *t*_*c *_approaches zero in the absence of phonon thermal conductance. When the phonon contribution dominates the thermal conductance of the SCQD junction, the optimization of ZT can be obtained by the thermal power defined as *S*^2^*G*_*e*_. We also found that the presence of electron Coulomb interactions can lead to either reduction or enhancement of ZT, depending on whether the Fermi level is below or above the QD level.

## Competing interests

The authors declare that they have no competing interests.

## Authors’ contributions

DMTK and Y-CC established the theoretical formalism. DMTK carried out the numerical calculations and drafted the manuscript. Y-CC conceived this study and participated in its coordination. All authors read and approved the final manuscript.

## Authors’ information

DMTK received his Ph.D. degree from National Taiwan University in 1996. He joined the Department of Electrical Engineering, National Central University in 2003 as an assistant professor, became an associate professor in 2005, and professor in 2008. His main research interests include nanodevices and quantum transport.

Y-CC received his Ph.D. degree from the California Institute of Technology in 1980. He joined the Physics Department, University of Illinois at Urbana-Champaign in 1980 as a visiting research assistant professor and became an assistant professor in 1982, associate professor in 1986, and professor in 1991. In 2005, he joined Academia Sinica, Taiwan as a Distinguished Research Fellow of the Research Center for Applied Sciences. His main research interests include condensed matter theory, semiconductor electronics, photonic materials, and optoelectronic devices.
